# Cultivating Health in All Policies Mindsets: An Ongoing Journey to Integrate Health and Housing in Georgia

**DOI:** 10.3390/ijerph21121639

**Published:** 2024-12-10

**Authors:** James E. Dills, Margaret E. Major, Michelle J. Marcus, Taylor S. Williams, Leigh Alderman

**Affiliations:** Georgia Health Policy Center, Andrew Young School of Policy Studies, Georgia State University, P.O. Box 3992, Atlanta, GA 30303, USA; mmajor6@gsu.edu (M.E.M.); mmarcus2@gsu.edu (M.J.M.); twilliams318@gsu.edu (T.S.W.); lalderman2@gsu.edu (L.A.)

**Keywords:** affordable housing, health equity, cross-sector collaboration, health in all policies, health impact assessment, systems thinking, vital conditions

## Abstract

This project report explores the use of a Health in All Policies (HiAP) approach by the Georgia Health Policy Center (GHPC) to integrate health perspectives into affordable housing policy and practice in Georgia. It focuses on five interconnected projects from over a decade-long collaboration to illustrate how the GHPC team started with a Health Impact Assessment (HIA) to seed cross-sector partnerships and then sustained them through subsequent collaborations. These projects included comprehensive-, intermediate-, and rapid-scale HIAs, as well as direct collaborations on housing development funding applications and a multidisciplinary research study on public housing renovations. This paper documents how HiAP tactics were applied across these projects to foster sustained collaboration and promote health equity. The insights offered highlight how the HiAP approach cultivated mindset shifts among public health practitioners, housing stakeholders, and policymakers, leading to a broader understanding of health and housing intersections. This synthesis contributes practical guidance for practitioners looking to advance the Vital Conditions for Health and Well-Being through housing policy initiatives.

## 1. Introduction

Over the past thirty years, public health professionals have increasingly recognized the need to collaborate with other sectors to address the root causes of poor health outcomes and inequities [1]. Public health practitioners have traditionally referenced these root causes as the social determinants of health (SDOH) [2,3,4]: however, the practice is evolving to reframe SDOH as Vital Conditions for Health and Well-Being (Vital Conditions), a more approachable framing for non-health collaborators [5]. The intersections between housing and health are increasingly recognized as critical in shaping the well-being of communities, and as a result, “humane housing” is designated as one of the seven Vital Conditions [5,6]. As public health practitioners seek to improve health and advance equity by informing decision-making systems that affect the distribution of Vital Conditions, a holistic and collaborative approach like Health in All Policies (HiAP) becomes essential [7]. This paper explores a decade-long application of the HiAP approach within Georgia’s affordable housing system. It reflects on a collaborative journey through five interrelated projects, how common HiAP tactics were applied therein, and what insights emerged for practice in pursuit of general HiAP goals established in the research literature [8,9].

### 1.1. Housing as a Vital Condition

Housing is widely acknowledged as a fundamental determinant of health [10]. This Vital Condition impacts health through multiple pathways, including access to safe and stable living environments, proximity to resources like education and healthcare, and the psychosocial benefits of secure housing [6]. Substandard housing conditions have been linked to a range of adverse health outcomes, from respiratory illnesses [11] and cardiovascular disease [12] to mental health issues [13] and infant mortality [14]. Public health practitioners have long used a variety of methods to build awareness of the significant influence housing conditions have on well-being [15]. However, these practitioners rarely create housing policy directly, nor do they typically have active roles in the physical development of housing projects. Informing these integral components of the housing system requires public health practitioners to explore different ways to approach working with their counterparts in the housing sector.

### 1.2. Health in All Policies

Health in All Policies (HiAP) is a collaborative approach that seeks to integrate considerations of health, well-being, and equity into decision-making processes and achieve mutually beneficial goals across sectors, including housing, transportation, and education (to name a few) [16]. The American Public Health Association (APHA) identifies the five overarching goals of the HiAP approach as promoting health, equity, and sustainability; supporting intersectoral collaboration; benefiting multiple partners; engaging stakeholders; and creating structural or procedural change [9]. A review of HiAP implementation in the United States identified seven broad tactics evident in practice to support these goals: developing and structuring cross-sector relationships; incorporating health into decision-making processes; enhancing workforce capacity; coordinating funding and investments; integrating research, evaluation, and data systems; synchronizing communications and messaging; and implementing accountability structures [8]. In the context of housing, HiAP offers a framework for embedding health perspectives into housing policies and practices, ensuring that decisions made in the housing sector contribute to the health and well-being of communities.

HiAP can take many forms and includes a spectrum of activities and collaborative efforts that range from one-off engagements to ongoing partnerships in which agencies have formal agreements to integrate considerations of health into their decision-making processes [9]. HiAP has been adopted by many countries as a policy at the national level [17]. Within the United States, public health practitioners at local, state, and federal levels have successfully used a HiAP approach to encourage stable, healthy, and affordable housing through a variety of programs and policies [18]. However, these successes have largely been documented as individual case reports or program evaluations and have not been disseminated broadly [19,20]. Despite awareness of the value of a HiAP approach and the accessibility of the common tactics that support it, public health practitioners face multiple challenges to effective and sustained collaboration with housing decision-makers [20,21,22].

### 1.3. Challenges for Practice

Decision-making within the affordable housing system is based on a series of complex factors and a variety of stakeholders, often not readily visible or known to public health practitioners interested in leveraging the power of safe, stable, and affordable housing to advance health equity and well-being. Public health practitioners have difficulty identifying how to intervene in the housing sector and often lack the capacity (time and knowledge) to develop the relationships needed for a HiAP approach. Similarly, practitioners in the affordable housing system are often unaware of public health approaches to promote well-being and equity and may be unaware of how to leverage public health data and knowledge to improve the health and well-being of people who will live in the homes they work to build and maintain. Implementing a HiAP approach also faces risks of being viewed as health imperialism [22]—promoting health at the cost of devaluing the housing industry’s priorities.

Much of the literature on HiAP to date has focused on how governmental public health agencies—local, state, and federal health departments—have initiated or facilitated the integration of health into decision-making in other government sectors, but less has been written about non-governmental actors using a HiAP approach to address the upstream drivers of health and well-being [8,17,19,20,23]. Learnings from over a decade of cross-sector work between a public health institute and decision-makers in the affordable housing system in Georgia suggest that these challenges can be overcome and that mutually beneficial outcomes, including new learning opportunities for public health practitioners, can emerge through adaptive and ongoing collaboration.

### 1.4. Report of One Team’s Approach to Integrating Housing and Health Perspectives: From HIA to HiAP

This report describes the ongoing journey of a team of public health researchers at the Georgia Health Policy Center (GHPC) in the Andrew Young School of Policy Studies at Georgia State University, who are using a HiAP approach to integrate health perspectives into affordable housing policy and practice in the state of Georgia. This study documents five HiAP projects that developed over time from an initial Health Impact Assessment (HIA) and discusses insights from this ongoing collaboration. Each project is summarized using relevant examples of the seven common HiAP tactics to ground practice in the existing literature and to reflect on how these strategies created additional opportunities for collaboration with new and existing stakeholders over time. The aim of this paper is to illustrate how these five interrelated projects generated insights for practice that cultivate HiAP mindsets and advance broad HiAP goals. The project descriptions and discussion will achieve the following:

- Explain how HIA created entry points for building partnerships and laid the foundation for sustaining collaborative relationships between public health practitioners and affordable housing stakeholders through the application of a HiAP approach;
- Describe how sustaining this HiAP approach contributed to important mindset shifts that led affordable housing practitioners and policymakers to more intentionally leverage public health perspectives to promote health and well-being in the communities impacted by their decisions;
- Identify how the HiAP approach contributed to mindset shifts in the GHPC team, promoting a more informed understanding of affordable housing practice that supported deeper collaboration and trust across sectors;
- Share key learnings from the GHPC’s continued efforts to shape affordable housing policy and practice in Georgia for the benefit of others interested in using a HiAP approach to better align housing and health perspectives in their communities.

Within this manuscript, the phrase “housing practitioner” refers to people who work primarily within the housing realm, ranging from decision-makers within state housing finance agencies like Georgia’s Department of Community Affairs (DCA) to leadership and staff of Public Housing Authorities at the local level. Housing practitioners also include private sector housing developers, designers, managers, consultants, and service providers. The term “public health practitioner” is similarly used as shorthand for a range of individuals focused primarily on public health perspectives, including state and local agency representatives as well as researchers, service providers, and health advocates.

## 2. Materials and Methods

To achieve the aims of this paper, the authors reviewed documentation from five projects representative of the GHPC team’s efforts to support the intentional integration of health perspectives into affordable housing policy and practice since 2013. The projects include three HIAs—one each at the comprehensive, intermediate, and rapid scales of this foundational HiAP tool [8,20]—and two HiAP efforts representing deeper integration of health perspectives into work with both public and private sector affordable housing partners. The project descriptions focus on how each collaboration illustrated basic components of HiAP practice and informed subsequent opportunities to continue cross-sector learning (Table 1).

A retrospective review of project documentation was conducted across five key initiatives, selected for their role in advancing HiAP goals within the affordable housing sector in Georgia. This review adapted qualitative methods of document review and solicitation of experiential expert knowledge by compiling available public-facing and internal project documentation, followed by collaborative sense-making with GHPC team members involved with each project [24,25,26]. The team used seven common HiAP tactics, identified in the literature contemporaneously with the start of GHPC’s learning journey [8], to guide collaborative reflection leading to project descriptions. Each project was framed within one or two of these tactics, highlighted in the project description headings. This methodological approach enabled a systematic description of each project to inform team discussions that synthesized contributions to broader HiAP goals established by the APHA and the authors’ own insights for HiAP practice framed by those goals. Given their broad applicability, these tactics and goals from the literature are used for framing the collective insights from GHPC’s efforts across multiple projects within this paper. However, these tactics and goals only informed the GHPC team as they designed project-specific aims and were not explicitly part of project design. Those project-specific goals and strategies were too context-specific for use in this paper, which aims to synthesize learning across projects to generate insights applicable for HiAP practitioners across a range of settings.

While this approach facilitated a practical and contextually informed narrative, it relied primarily on the availability and comprehensiveness of project documentation rather than a more formalized coding or analytical framework. The limitations include potential biases in interpreting documentation and team reflections, as these are influenced by the perspectives of those involved in the projects. This method reflects an iterative, practice-based process rather than a rigorous qualitative methodology, and the findings should be interpreted with this context in mind.

## 3. Results

### 3.1. The Beginning of Cross-Sector Relationships and Integrating Research, Evaluation, and Data Systems: Comprehensive HIA of Low-Income Housing Tax Credit Policy

***Project Title: A Health Impact Assessment of the 2015 Qualified Allocation Plan for LIHTC in Georgia [27]|2013–2015***

This multi-year HIA funded by the Health Impact Project (a collaboration of the Robert Wood Johnson Foundation and The Pew Charitable Trusts) helped the Georgia Department of Community Affairs (DCA), the state housing finance agency, integrate health perspectives into the scoring rubric that determines the allocation of federal tax credits for developing affordable housing in the state. This initial collaboration with the DCA demonstrated the value of elevating public health perspectives in their existing work and led to years of subsequent project collaborations and thought partnership between the GHPC and affordable housing stakeholders in Georgia.

The U.S. Department of Housing and Urban Development states that “the Low-Income Housing Tax Credit (LIHTC) program is the most important resource for creating affordable housing in the United States today” [28]. A Qualified Allocation Plan (QAP) is the federally mandated process through which states issue these credits to qualified applicants, who in turn develop new or renovated affordable housing sites. In Georgia, the DCA updates the QAP annually, providing regular opportunities for engagement with individuals and organizations representing a range of perspectives relevant to community development in the state. The HIA facilitated more intentional inclusion of health perspectives, both in the process and in the resulting policies. The success of this initial approach led the Bipartisan Policy Center, a national non-profit organization not involved in the project, to note that with respect to maximizing health through the LIHTC policy, “Georgia is leading the way, and other states should follow suit” [29].

Relationships with state policymakers at the DCA initiated through the HIA process proved foundational to enhancing the GHPC’s understanding of the affordable housing system in the state and sustaining meaningful collaborations throughout subsequent projects. Conversations with DCA staff lead to buy-in from their leadership for the GHPC’s HIA project proposal and resulted in a successful grant application. Early in the HIA process, the project team assembled a Steering Committee of eighteen individuals with a range of perspectives on affordable housing and community development. The committee included several decision-makers from the DCA, representatives of the developer community, subject matter experts, and advocates for social justice. The HIA project team consulted with this group regularly throughout the process and had them vote on critical decisions like the overarching health issues on which the HIA would focus. Many individuals and the organizations represented on the HIA Steering Committee maintain collaborative relationships with the GHPC team and have been involved in their subsequent health and affordable housing projects.

By working with this group of housing stakeholders, the GHPC team grew an understanding of how the policy takes shape each year, where information from other sectors could be incorporated, and what doing so might mean for the housing developers whose funding applications are subject to the QAP. The HIA recommendations for incorporating data platforms from other state agencies into the QAP scoring rubric illustrate the evolution of this understanding for the GHPC team.

In one instance, the HIA recommended adjusting the QAP scoring aimed at siting affordable housing development in areas of higher opportunity for future residents by incorporating Georgia Department of Public Health (DPH) data that characterize local conditions through an index of over two dozen indicators. The DCA and other stakeholders recognized the potential value in using this index to enhance existing scoring, which focused on the poverty rate as the primary indicator of opportunity. They also recognized that doing so would be a significant shift in the policy, and applicants would need more time to fully understand the rationale behind the change before adopting it into their practice. As a result, the DCA chose not to implement that specific HIA recommendation in the 2015 policy, but they did indicate willingness to continue exploring ways to incorporate this DPH data source into future iterations of the QAP and eventually adopted this recommendation in the 2016 version [30].

In another instance, the DCA readily adopted HIA recommendations to incorporate Georgia Department of Education (DoE) data into the 2015 scoring policy. In this case, the proposed change was conceptually more straightforward, improved upon an existing effort by the DCA to account for educational opportunity near proposed housing locations, and streamlined the process for developers to include relevant information in their applications. The DoE developed the College and Career Ready Performance Index (CCRPI) for K-12 schools as a comprehensive school improvement, accountability, and communication platform for all educational stakeholders that promotes college and career readiness for all Georgia public school students. The HIA recommended that the DCA “use the CCRPI to determine the quality of schools near proposed development sites and provide scoring incentives for locating in the attendance zones of above average schools” [27]. This change was included in the 2015 QAP under the “Quality Education Areas” section of the scoring criteria. It offered a more straightforward process than the educational criteria first introduced in the 2014 QAP, which required applicants to perform their own time-consuming calculations based on test scores by school and grade to determine if nearby schools met the quality threshold required for their project to receive points. With the use of the CCRPI in 2015, applicants could include a more robust measure of school quality than test scores alone and could do so more quickly using data readily available for each school on the DoE’s CCRPI website.

Incorporating firsthand perspectives of current LIHTC residents presented a challenge to the team, especially at this early stage of collaboration, where a primary aim was to build relationships with decision-makers and housing practitioners. The scoping phase of the HIA considered tradeoffs between incorporating resident perspectives through the inclusion of state-level advocacy organizations and investing resources in direct engagement with residents. The group collectively decided on the former, believing that conducting the latter well for a statewide project would exhaust the project’s limited resources and leave little remaining to invest in developing actionable recommendations for agency decision-makers, which was the purpose of the HIA. Future projects continued to struggle with this challenge for various reasons until resources were secured specifically for the purpose of direct resident engagement.

This HIA helped the GHPC team learn firsthand how the DCA balances input from the range of perspectives that seek to inform their annual QAP update, striving for continuous improvement while at the same time limiting disruptions to the application process for developers from year to year. By both conveying why public health perspectives are relevant to the DCA’s goals for affordable housing development and offering options for how to actionably incorporate those perspectives into existing processes, the GHPC team demonstrated their willingness to be a thoughtful partner in two-way learning and collaboration with the DCA and other stakeholders at the intersection of housing and health.

### 3.2. Synchronizing Communication and Messaging: Intermediate HIA of Three Affordable Housing Developments

***Project Title: Georgia Affordable Housing Health Impact Assessment [31]|2016***

In 2016, the GHPC conducted a second affordable housing HIA, this time on behalf of the DPH, with funding from the U.S. Centers for Disease Control and Prevention. The intermediate-scale HIA examined three specific housing development plans that received tax credit financing under the 2015 QAP. The results helped housing developers consider how their siting, design, and operational decisions could be more supportive of community health. The GHPC team and representatives from the DPH advanced their understanding of where in the housing development process public health input could be most impactful on housing partners’ decision-making. The HIA findings also led to further changes in state-level affordable housing policy through the QAP, including the adoption of the 2015 recommendation to use DPH data on community context described above.

This HIA contributed to a subtle but impactful synchronization of communication between health and housing development stakeholders wherein decisions about siting, design, and operations emerged as a strong organizing frame for characterizing collaboration points between the two sectors. While these concepts were not necessarily new to participating partners, the insights generated through the HIA process helped reconcile “upstream–downstream” frames commonly used by public health practitioners with the realities of housing development practice and timelines [32].

Where a housing development is sited (located) determines how connected residents are to the communities in which they will live—ideally to amenities that advance well-being and health equity. The HIA stakeholders recommended a focus on aspects of the state’s QAP rather than individual developer siting decisions as a more upstream (or systemic) opportunity for incorporating health perspectives into housing development siting. While siting recommendations were not made for the three study projects within this HIA because their locations were already selected, the assessment provided evidence that changes to the QAP made as a result of the 2015 HIA (e.g., the use of CCRPI data referenced above) helped incentivize siting in areas likely to be more supportive of resident health, as indicated by research evidence compiled within this and the prior HIA.

Design decisions correspond with the “mid-stream” level of public health intervention, and while many emerging best-practices for healthy housing design exist, the HIA results found that these were difficult decisions to influence for the three developments involved. Participating housing stakeholders attribute this difficulty to the amount of preliminary design work development teams conduct prior to submitting their LIHTC applications. For health perspectives to substantially inform housing design decisions, they must be included much earlier in the development timeline than was possible in this project. Only one of the three was able to implement a site design recommendation: including a community garden as part of redesigning landscape plans to better comply with on-site stormwater management practices required for certain sustainable building certifications. The alignment with environmental sustainability practice revealed an opportunity for a better understanding of how healthy design strategies can be incorporated into existing processes with which development teams are already familiar.

Housing operations include management practices, types of programming or services offered on site, and the relevant partnerships needed to deliver those programs. Decisions about these aspects of housing correspond to “downstream” types of interventions for public health practitioners and were most amenable to changes informed by the HIA. Providing developers with timely and easily understandable data on the health concerns of the surrounding community and/or the potential tenant population allows them to more finely tailor service offerings to the needs of residents. Implementing this type of approach also fosters new partnerships at the local level between developers, property managers, housing service coordinators, and local, community-based initiatives to improve health.

This HIA proved a timely opportunity for collaboration, coming directly after the completion of the 2015 HIA project. Several of the same DCA stakeholders continued to be involved, as well as representatives from the three specific developments being examined. Bringing developers more deeply into the collaboration helped public health stakeholders better understand housing partners’ mindsets about their role in promoting healthy communities through the provision of affordable housing units. Policy changes informed by this HIA directly led to the GHPC’s next collaborative project with the housing sector.

### 3.3. Implementing Accountability Structures: Healthy Housing Initiative Consultations

***Project Title: Healthy Housing Initiative Consulting (Multiple Instances) [33]|2017–2020***

Directly informed by prior collaborations with the GHPC and other health stakeholders, the DCA included an optional scoring section in the 2017 QAP for developers to propose “Healthy Housing Initiatives” as part of their applications. This new application component produced an incentive for developers to seek external consultation from public health experts to assist them in designing initiatives that would meet policy requirements. The GHPC served as a consultant for multiple development applications and in doing so continued to grow the team’s understanding of developer practices and how DCA policy influences them.

Based largely on the approaches explored in the 2016 HIA and ongoing thought partnership with the GHPC, the DCA identified health outcomes for residents as the second of six state priorities for allocating LIHTC resources in the 2017 QAP. The DCA defined this priority using the following language in all subsequent QAPs through 2023:

“Health Outcomes for Residents: Physical and mental health are necessities for thriving individuals and families. The location where a household lives strongly influences household health through things like access to quality care, education, and healthy foods. In addition, safe, quality affordable housing provides the foundation and central location for encouraging healthy lifestyles. As such, the DCA has a strong commitment to encouraging better health outcomes for residents through site selection, site design, community partnerships, and focused services” [34].

Developers were held accountable to this explicit priority through a QAP scoring section designed to incentivize “Healthy Housing Initiatives (HHIs)”. As defined in the 2017 QAP, “A strong Health Initiative will leverage available resources by working with health agencies, programs, non-profits, or civic groups to maximize community services, technical service and financial support for healthy living programs by establishing partnerships to leverage services that benefit health. Applicants are encouraged to target healthy initiatives to local community needs” by using health data from the Georgia Department of Public Health, the County Health Rankings & Roadmaps, and Community Health Needs Assessments. The policy goes on to stipulate that the plan for each initiative includes a “proposed strategy for measuring outcomes” [34].

The GHPC contributed to implementing this accountability structure through a partnership with a Georgia-based sustainable construction firm, who already provided application preparation services for developers seeking sustainability certifications. The firm contracted with the GPHC team to offer HHI consulting services along with their existing sustainability services for development teams. Over a four-year period, the GHPC consulted with six different developers to design health initiatives for twenty-five tax credit applications. Each involved an examination of local health data to drive the selection of environmental design and service programming options to support resident health and well-being, along with brokering relationships between development teams and local health partners (e.g., health care systems, local clinics, pharmacies, etc.). Based on the proportion of successful tax credit applications, the GHPC directly informed the design and implementation of health-supporting strategies for over 1000 housing units across multiple communities and projects in Georgia. Examples of these strategies include adding small community gardens to preliminary designs, providing nutrition education opportunities for residents through the University of Georgia Cooperative Extension system, and hosting regular on-site wellness checks for residents by local health care providers.

Though working earlier in the development timeline than with previous projects, the GHPC’s role with HHI consulting evolved to be relatively limited in nature and challenged their initial vision of mutual learning across sectors. After the first year with these projects, the GHPC team recognized that even if development teams had interest in learning about conceptual connections between their housing work and community health, their timelines did not readily allow for it. Thus, the GHPC streamlined their approach to deliver exactly what the development teams needed to submit their LIHTC applications by the DCA deadline each year. Mutual learning became a more implicit or passive goal of these consultations, while other cross-sector engagements remained explicitly focused on learning and supplied space for that to actively happen.

### 3.4. Continued Incorporation of Health into Decision-Making Processes: Rapid HIA of Proposed Recommendations for Mitigating Air Pollution Exposure

***Project Title: Near-Roadway Air Quality (NRAQ) and LIHTC in Georgia-Student-led Rapid HIA [35]|2019***

In 2019, the Region IV Public Health Training Network’s Pathways to Practice Scholars Program supported a public health graduate student to complete a practicum experience with the GHPC based on an interest in the intersection of environmental health concerns and housing policy. The practicum produced a rapid-scale HIA that revisited an unadopted recommendation from the 2015 HIA and provided the GHPC team with an opportunity to convene several affordable housing stakeholders around a specific public health issue: exposure to air pollution from high-traffic roadways.

Based on strong public health evidence, the 2015 QAP HIA recommended that the DCA “Reduce potential exposures to air pollution by adjusting scoring criteria to incentivize development in locations farther than 200 m (650 feet) from roadways carrying more than 25,000 vehicles per day”. The recommendation was ultimately not adopted, with the original HIA noting that it “was included in the draft 2015 QAP but removed from the final version due to developer concerns about reduced property visibility on lower traffic roads affecting marketability. In response, solutions that balance mitigation of potential exposure to pollution and project visibility should be further explored. Examples might include increasing the threshold to 50,000 vehicles per day, designing sites to have residential buildings set further back from the busiest roadways, or planting evergreen trees to filter pollution”.

The opportunity for a rapid HIA several years later provided space for the GHPC to provide additional data and further explore housing stakeholders’ mindsets around this aspect of development siting and design. The rapid HIA included a literature review of air pollution exposure and high-traffic roadways, a geospatial analysis of LIHTC property locations respective to high-volume roadways from 1988 through 2018, and a convening with several affordable housing developers and sustainable building experts—all of whom had been involved with the GHPC’s previous housing and health efforts. The presentation included background on the original recommendation from 2015, NRAQ health and economic impact literature, and the main findings that ~26% and ~10% of LIHTC developments fall within 500 m and 200 m of major roadways (≥25,000 Annual Average Daily Traffic), respectively. The preliminary policy recommendation targeted developments within a 500 m buffer and identified sections of the DCA environmental manual that could be useful in addressing this issue. Developing a further understanding of potential NRAQ mitigation strategies was cited as the most reasonable action to take moving forward.

Though policy changes did not result from this project, the GHPC continued to provide new and relevant health information to the QAP development process based on the work. Stakeholders expressed continued willingness to examine the issue but wanted to better understand how current green building standards might mitigate exposure risk in these locations and how to characterize tradeoffs between the risk of resident exposure to pollution and the potential benefit of access to community amenities. The experience of this project highlights how creating opportunities for collaborative problem-solving and exploration helps sustain relationships and can grow an understanding of how stakeholders with different perspectives approach an issue, even when the goal of policy change is not achieved.

### 3.5. Coordinating Investment and Enhancing Workforce Capacity: Homes for Healthy Futures Project

***Project Title: Georgia Homes for Healthy Futures [36]|2017–2025 (ongoing)***

The partnerships between the GHPC, the DCA, and other affordable housing stakeholders had matured enough by 2017 to advance their cross-sector collaborations to a new level through the Georgia Homes for Healthy Futures (HHF) project. This effort created space for continued learning and application by existing partners and capacity building for new ones and included greater opportunity for resident involvement.

The Kresge Foundation funded the HHF project, a partnership between the DCA, the GHPC, the Southface Energy Institute (a new partner for the GHPC), and six local Public Housing Authorities (PHAs) around the state. In addition to grant funding from Kresge, housing developers also provided funding to support participation in the initiative, a critical step toward more sustainable mixes of funding and an indicator of housing stakeholders recognizing the value of collaboration with the public health sector and HiAP perspectives. This five-year initiative (subsequently extended to accommodate pandemic-related disruptions) focused on building capacity, knowledge exchange, and outcome tracking across diverse settings around the state to ensure that public housing communities are healthy places. The GHPC leveraged previous experience integrating health perspectives into housing policy (i.e., the QAP) and practice (i.e., the 2016 HIA and HHI consulting) to provide evidence-informed recommendations on public housing renovation plans and gather resident input on those plans. HHF included resources for collecting and analyzing primary and secondary data to evaluate the work’s process, impact, and outcomes—a component lacking in previous efforts.

By recording and accounting for local contexts across different PHA sites, the GHPC sought to illuminate what works for whom, and under what circumstances, to help ensure replicability in various contexts and communities. To build capacity across the system, the GHPC designed, facilitated, and evaluated a Housing and Health Learning Academy series that included participants from PHAs, redevelopment teams, local Public Health Departments, local service providers, resident leaders, and other perspectives. One of the first outputs from the Learning Academy was a collaboratively developed definition of “Homes for Healthy Futures” as a holistic framework that supports sustainable systems and practices that intentionally focus on achieving environmental, individual, and community aspirations, leading to thriving people and places now and for generations to come. Each of the first three Learning Academy sessions had over 30 participants, representing all the participating communities and a range of perspectives. Session evaluations demonstrated that nearly all participants (over 90%) agreed that they (1) had a greater understanding of the intersection of sustainability, health, and housing and (2) were able to develop or strengthen relationships with other participants in the workshops.

In addition to providing health and sustainability input on multiple public housing renovation plans, this project also allowed the GHPC team to work directly with residents impacted by those renovations. A challenge to engaging residents in earlier collaborations was that those projects were focused on policy, design, and operation decisions impacting future residents of housing units yet to be placed in service. The HHF project was the first with a focus on housing renovations that impacted existing residents. Two specific resident engagement opportunities are worth mentioning here. First is the participation of residents in community approaches to collect data both before and after renovations through health assessment of environmental and programmatic changes at each site. Secondly, resident leaders participated with other community stakeholders in the Learning Academy.

The HHF project is ongoing as of the writing of this manuscript and continues to present the GHPC team with opportunities to facilitate cross-sector learning and knowledge exchange. Team members are currently analyzing project evaluation data to characterize impacts both on residents’ health behaviors and on the housing practitioners’ decision-making processes and mindsets.

## 4. Discussion and Insights for Cultivating a HiAP Mindset

This section illustrates how cultivating a HiAP mindset can be advanced by employing established methods, such as HIA, alongside strategic approaches to integrating health considerations into affordable housing policy and practice. A HiAP mindset is characterized by a willingness to collaboratively grow an understanding of cross-sector perspectives in pursuit of systemic actions that tune policy and practice toward more equitable outcomes. The following synthesis reflects on how the application of seven common HiAP tactics across the five projects described above coalesced in an ongoing learning journey that advances overarching HiAP goals outlined by the APHA [9]. While recognizing limitations inherent in the retrospective and localized context of this review, this synthesis provides valuable insights for practitioners seeking to promote the Vital Conditions for Health and Well-Being through sustained cross-sector efforts in their communities. These insights will be particularly useful for practitioners in the U.S. looking to start or continue their own health and housing collaborations because all states (and some other jurisdictions) have a QAP for LIHTC, which is the high-leverage policy where the GHPC began the journey that generated these insights.

### 4.1. Promoting Health, Equity, and Sustainability

***Insight: successfully advancing population health through collaboration with other sectors requires adopting sector-specific terminology and frames, ensuring public health goals are integrated without overshadowing those sectors’ existing priorities.***

For collaborative work that aims to promote public health through changes in housing policy and practice, centering housing practitioners’ perspectives rather than those of the health sector can underpin relationship building and opportunities for mutual learning. Meeting housing practitioners where they are in terms of the health, equity, and sustainability outcomes important to them allowed the GHPC team to more readily demonstrate the value of collaboration with public health practitioners. It also allowed the public health practitioners to learn more about how broad terms like health, equity, and sustainability carry meaning in another sector, leading to an integration of perspectives between sectors rather than an unintended imposition of health-focused ones.

From the early stages of collaboration in the 2015 HIA, the GHPC team observed that health, equity, and sustainability were already important concepts to housing partners. For them, compliance with building safety codes and attaining environmental sustainability thresholds for specific certifications drives their perspective on health. Positioning housing as central to a wider view of promoting thriving communities better aligns with overarching goals for community development that often guide affordable housing practice.

Strategically positioning HiAP efforts within the frames of the housing sector also facilitated mutual learning that expanded perspectives. Through capacity-building efforts like the HHF Housing and Health Learning Academy, the GHPC team was able to collaborate with housing practitioners to broaden what health, equity, and sustainability mean to one another and to others in the system like residents, service providers, and local governments.

The key for public health practitioners is to be comfortable letting the explicit promotion of public health views of health, equity, and sustainability be secondary to growing their understanding of how other sectors talk (and think) about these topics—at least in the early stages of collaboration. The GHPC’s experience here, particularly in the 2016 HIA and HHI consultations, demonstrates how adopting the housing sector’s framing of siting, design, and operational decision points allowed for greater nuance in the packaging of health-informed recommendations, ultimately making them more useful for housing decision-makers. This housing-centered, relationship-building, and incremental approach helped expand the understanding of housing’s role in promoting health, equity, and sustainability in the state. This expanded understanding led to a mindset shift for housing decision-makers, evident in the DCA adopting resident health as an official priority in statewide housing policy.

### 4.2. Supporting Intersectoral Collaboration

***Insight: discrete projects with structured approaches create space for growing and sustaining collaborative relationships.***

As a Vital Condition, humane housing readily connects to a range of other conditions that collectively set the context for a community’s health. This connection leads to an abundance of collaboration points for health and housing practitioners throughout the system. Strategic approaches to cross-sector partnership can sustain these collaborations as part of a longer-term journey to cultivate HiAP mindsets—from both health and housing perspectives. Early in this journey, developing discrete projects facilitated collaboration, allowing stakeholders to explore and address common challenges in affordable housing. For the public health practitioners, these early projects enlightened them about the policies and processes housing practitioners encountered every day. Equipped with a deeper understanding of these structures, the GHPC focused subsequent project efforts on topics that were salient for their collaborators in the housing sector. This strategic approach demonstrated a collaborative mindset that contributed to the trust needed for longer-term partnerships.

Initiating collaboration through an HIA supported mutual learning with an established HiAP tool. The structure of the HIA process is adaptable and inclusive. Applying it to a state-level policy that contains numerous avenues for cross-sector analysis and input like the QAP proved strategic for convening an advisory group that represented a range of perspectives on the housing and health issues at play. HIA allowed this group to decide which specific topics would be analyzed and, more importantly, which aspects of decision-making were most receptive to shifts suggested by HIA recommendations. The successful adoption of several HIA recommendations demonstrated the value of this approach, as did the agreement of stakeholders to participate in the next project, another deployment of the HIA tool, focused this time on three specific housing developments rather than state-level planning. By continuing the journey, this time equipped with the collective experience from the first HIA, collaborators began to express a shared mindset around the value of health perspectives for affordable housing practice.

Subsequent projects continued to create opportunities for mutual learning across sectors, fostering a HiAP mindset and critical partnerships that continue to thrive within the affordable housing system in Georgia. Building these relationships takes time and adaptability. Having a series of projects within which collaboration takes place, focused on specific aspects of housing policy and practice, underpins the relationships needed not just for the development of a HiAP mindset but also for its sustainability.

### 4.3. Benefiting Multiple Partners

***Insight: sustained success is achieved through a deep understanding of perspectives and practices across sectors, which takes time but yields lasting mutual benefits that reinforce a HiAP mindset.***

Understanding the perspectives of different partners—such as affordable housing developers, policymakers, health departments, and local service organizations—helps foster effective and mutually beneficial approaches. The accumulation of these benefits grows and reinforces the HiAP mindset among collaborators.

The GHPC identified several benefits for different types of partners involved in their housing and health efforts. Policymakers gain valuable insights on structuring processes that support developers while ensuring benefits for communities. Developers, in turn, benefit by securing points in the QAP scoring system, making their projects more competitive. Communities benefit from improved housing and access to services that promote health and well-being. Additionally, health stakeholders, including health departments and local health organizations, benefit from the integration of health-promoting elements into housing policies and practices, leading to improved public health outcomes. Across all perspectives, the deeper involvement of health practitioners in affordable housing policies and practices can lead to mutually reinforcing benefits. Ongoing scholarship by the GHPC team aims to more fully characterize and analyze these mutual benefits, particularly as part of HHF project evaluation. Notably, the partnerships strengthened along the HiAP journey were a critical factor in securing the resources needed to support this scholarship, which is anticipated to produce findings that will benefit both the housing and the public health partners involved.

A HiAP mindset can also lead to mutual benefits through leveraging the intersection of multiple Vital Conditions. Along with basic needs for health and safety (health) and humane housing (housing), lifelong learning (education) is also included among the Vital Conditions. In the 2015 HIA, the GHPC identified an aspect of the policy that aimed to incentivize affordable housing development near well-performing schools through specific scoring criteria. However, the GHPC learned that the existing process for determining school quality within the QAP was particularly onerous for the housing practitioners developing tax credit applications. The HIA produced recommendations that restructured this QAP section to continue incentivizing educational access for affordable housing residents but to do so using an existing Department of Education data source that was at the same time more comprehensive in its metrics of school quality and more efficient for housing development teams to use. This relatively small change to the housing policy promotes benefits across housing, education, and health, better aligning practice and impact for three of the seven Vital Conditions.

### 4.4. Engaging Stakeholders

***Insight: effective engagement with diverse stakeholders in the affordable housing system requires a strategic and adaptive approach, grounded in building trust and understanding perspectives, to foster sustained impact.***

Many different groups influence and are impacted by the affordable housing system. Engaging this breadth of stakeholders requires time, relationship building, and adaptability. Navigating the complexities of the system and working through key decision-makers or “gatekeepers” to certain stakeholders requires a level of trust between housing and health practitioners. The GHPC made strategic decisions early in their journey to intentionally build this trust within the system by focusing on growing their own understanding of housing decision-making processes and the perspectives of those within the housing policy and development space prior to direct engagement with community members and residents. Different contexts may have called for sequencing engagement differently. For example, if the 2015 HIA had focused on a single housing development in a specific community rather than on state-level policy change, then the team would have likely started the journey by building trust within that local community. Health professionals should strategically sequence engagement based on where they enter the other sector’s system and the resources available to support engagement activities from project to project.

In addition to being strategic, the GHPC’s approach to stakeholder engagement was also adaptive. Within the context of each project, there were specific engagement needs. The team adapted approaches based on those project needs while always recognizing which types of stakeholders were missing or underrepresented. This awareness helped identify opportunities for subsequent engagement during future phases of the collaborative journey. The 2015 HIA was focused on state-level policy, so stakeholder engagement focused on understanding the perspectives of those closest to decision-making about that policy (e.g., state agencies, state-level advocacy organizations, etc.). The 2016 HIA focused on specific development projects and presented the opportunity to more directly engage with those developers while continuing to nurture relationships with the state-level stakeholders from the 2015 project. Limited resources and the nature of the decisions being considered did not readily allow for direct resident engagement, which the team recognized as a gap to address in future efforts. Once resources were secured for the HHF project in 2017, the GHPC and their growing network of housing partners and collaborators were working in a context where direct resident engagement was more feasible. One reason for this feasibility is that the HHF project focuses on housing renovations rather than new development, so there were existing residents with whom to engage. Another was that the “gatekeepers” from the state agency and the development community had established the GHPC as a trusted partner to work with and elevate the voices of residents through the project.

The GHPC’s approach to understanding stakeholder perspectives allowed them to better comprehend the mental models, capacities, and needs of housing developers and policymakers, leading to more effective engagement. By recognizing where stakeholders were coming from, the team was able to introduce health perspectives into decision-making processes more smoothly. Engagement with stakeholders was viewed as a journey, where the initial focus on high-level policymakers set the stage for more thoughtful engagement with residents and other practitioners later. Gaining buy-in from decision-makers was critical for enacting changes that would ultimately improve health outcomes. As the project progressed, the team built the capacity to engage residents more effectively. The deeper their understanding of stakeholders’ needs and strengths became, the better they were equipped to influence upstream processes and practices that support overall health and well-being.

### 4.5. Creating Structural or Procedural Change

***Insight: systems thinking is essential for identifying, influencing, and implementing structural and procedural changes in non-health sectors, ensuring that common HiAP tactics are effectively adapted and sustained through project-specific contexts.***

The purpose of HiAP is to shift decision-making systems to be more inclusive of health perspectives so that non-health sectors more positively influence population health and equity. Structural and procedural changes indicate this shift in systems. The GHPC applied the critical skill of systems thinking throughout their HiAP journey to identify, inform, and implement changes within the affordable housing system. The ability to achieve this success stemmed from a deep commitment to learning about and understanding how the structures within that system are interconnected and underpin opportunities for community health and well-being.

Policies are key structural elements in the housing system. The GHPC succeeded in identifying the QAP as a critical policy that influences population health through its impact on the distribution and quality of affordable housing units in the state. They then worked with a variety of stakeholders over several years to understand and then inform changes to that policy through HIAs. Each project along the journey allowed the GHPC to participate in and thus inform the state’s policy development procedures, evident in annual listening sessions where health stakeholders share their perspectives with the DCA as the QAP is revised. In another part of the system, HHI consulting allowed the GHPC to participate in the implementation of some of the policy changes they informed, further growing their understanding of the system. By brokering developers’ relationships with local health service providers to serve future residents of their properties, they also informed procedures within specific housing developments. In the HHF project, the GHPC team and their partners have the opportunity to weave together multiple threads of learning from their shared journey, collectively identifying, informing, and implementing structural and procedural changes in collaboration with residents and a growing list of local stakeholders.

The GHPC’s ongoing and future work aims to more fully characterize how well changes to policies and practices in the affordable housing system informed by health perspectives are impacting opportunities for health in Georgia’s communities. The team is also focusing evaluation resources on continuing to grow an understanding of how housing stakeholders’ mindsets have shifted because of their participation in the HiAP journey.

## 5. Conclusions

This report demonstrates that initiating and sustaining cross-sector relationships between public health and affordable housing practitioners and policymakers can lead to mindset shifts that more intentionally leverage affordable housing as a platform for fostering well-being and health equity. It offers practical insights for future initiatives seeking to integrate health perspectives into other sectors’ decision-making systems. Three overarching takeaways emerged.

First, building cross-sector understanding is key. Successful HiAP implementation requires a mutual understanding of each sector’s priorities, processes, and perspectives. Public health practitioners must invest time in learning about the systems and mental models of non-health sectors in collaboration with stakeholders from those sectors. Applying approaches like HIA creates structured spaces for cross-sector learning. This effort builds trust and opens pathways for embedding health perspectives into decision-making processes, leading to long-term, impactful changes.

Second, effective stakeholder engagement is a journey that must be adapted to project-specific contexts. The GHPC’s approach demonstrates that the strategic sequencing of engagement—in their case, focusing first on decision-makers and then gradually involving community members—can strengthen collaborative efforts and ensure that health considerations are integrated into broader system changes. Flexibility in engagement strategies also helps address evolving project needs and the meaningful inclusion of underrepresented stakeholders.

Finally, systems thinking is essential. Applying systems thinking allows practitioners to identify leverage points where health perspectives can be meaningfully integrated into other sectors. By understanding how policies, practices, and processes are interconnected, collaborators can target changes that promote shared goals across sectors, such as sustainability, well-being, and equity. This approach is broadly applicable to work that addresses the Vital Conditions for Health and Well-being and can ensure that collaborative efforts are aligned with both sector-specific and public health priorities.

Future research should build on the insights offered in this project report to deepen the understanding of how cross-sector collaborations influence both community well-being and housing-related outcomes. Rigorous evaluations of projects and programs will be critical to measuring the short- and long-term impacts of integrating health considerations into housing decisions, particularly on resident health and systemic equity. Additionally, exploring the reciprocal influence of health-focused initiatives on housing-related outcomes, such as tenant retention, housing quality, and neighborhood stability, will provide valuable insights. Studies that investigate the mechanisms driving successful and sustained HiAP collaborations—such as trust-building strategies, adaptive funding models, and shared governance structures—will be especially beneficial for shaping the future of public health practice and advancing equitable housing policies.

## Figures and Tables

**Table 1:** Summary of five projects examined in this project report.

Project Title	Dates	Lead(s)	Funder	Main Objective	Approach
A Health Impact Assessment (HIA) of the 2015 Qualified Allocation Plan (QAP) for Low-Income Housing Tax Credits (LIHTC) in Georgia	2013–2015	Georgia Health Policy Center (GHPC) and Georgia Department of Community Affairs (DCA)	Health Impact Project (a collaboration of the Robert Wood Johnson Foundation and The Pew Charitable Trusts)	Integrate health into LIHTC policy through adjustments to QAP requirements and scoring	Comprehensive HIA
Georgia Affordable Housing Health Impact Assessment	2016	GHPC and Georgia Department of Public Health (DPH)	U.S. Centers for Disease Control and Prevention (CDC) Healthy Community Design Initiative	Examine three planned housing developments that received LIHTC financing under the 2015 QAP	Intermediate HIA
Healthy Housing Initiative Consulting (Multiple Instances)	2017–2020	GHPC and SK Collaborative	Housing developers	Support developers in designing health-focused initiatives	Secondary analysis of community health data analysis; designing potential health-promoting strategies and brokering relationships between development teams and local health service providers
Near-Roadway Air Quality (NRAQ) and LIHTC in Georgia	2019	GHPC	Health Resources and Services Administration (HRSA) Region IV Public Health Training Network	Revisit 2015 QAP HIA recommendation to examine potential air pollution impacts on LIHTC units	Student-led rapid HIA
Georgia Homes for Healthy Futures (HHF)	2017–ongoing	DCA, GHPC Southface Energy Institute, Local Public Housing Authorities, and Housing Redevelopment Teams	Kresge Foundation	Ensure that public housing renovations support healthy communities	Capacity building and knowledge exchange through Healthy Green Building Plans and a Housing and Health Learning Academy; pre–post renovation evaluation of environmental changes and residents’ health status and experience

Caption: this table lists each of the five projects described in this project report and includes summary information on the project period, leading organization(s), funding source, primary objective, and approach(es) employed for each.

## Data Availability

Data is contained within the article.
